# The Cancer Financial Experience (CAFÉ) study: randomized controlled trial of a financial navigation intervention to address cancer-related financial hardship

**DOI:** 10.1186/s13063-022-06344-3

**Published:** 2022-05-13

**Authors:** Nora B. Henrikson, Melissa L. Anderson, John Dickerson, John J. Ewing, Robin Garcia, Erin Keast, Deborah A. King, Cara Lewis, Blake Locher, Carmit McMullen, Consuelo M. Norris, Amanda F. Petrik, Arvind Ramaprasan, Jennifer S. Rivelli, Jennifer L. Schneider, Lisa Shulman, Leah Tuzzio, Matthew P. Banegas

**Affiliations:** 1 Kaiser Permanente Washington Health Research Institute, Seattle, WA USA; 2 Kaiser Permanente Center for Health Research, Portland, OR USA; 3 University of California San Diego, San Diego, CA USA

**Keywords:** Financial hardship, Cancer, Supportive care, Patient navigation

## Abstract

**Background:**

There is an urgent need for evidence on how interventions can prevent or mitigate cancer-related financial hardship. Our objectives are to compare self-reported financial hardship, quality of life, and health services use between patients receiving a financial navigation intervention versus a comparison group at 12 months follow-up, and to assess patient-level factors associated with dose received of a financial navigation intervention.

**Methods:**

The Cancer Financial Experience (CAFÉ) study is a multi-site randomized controlled trial (RCT) with individual-level randomization. Participants will be offered either brief (one financial navigation cycle, Arm 2) or extended (three financial navigation cycles, Arm 3) financial navigation. The intervention period for both Arms 2 and 3 is 6 months. The comparison group (Arm 1) will receive enhanced usual care. The setting for the CAFÉ study is the medical oncology and radiation oncology clinics at two integrated health systems in the Pacific Northwest. Inclusion criteria includes age 18 or older with a recent cancer diagnosis and visit to a study clinic as identified through administrative data. Outcomes will be assessed at 12-month follow-up. Primary outcomes are self-reported financial distress and health-related quality of life. Secondary outcomes are delayed or foregone care; receipt of medical financial assistance; and account delinquency. A mixed methods exploratory analysis will investigate factors associated with total intervention dose received.

**Discussion:**

The CAFÉ study will provide much-needed early trial evidence on the impact of financial navigation in reducing cancer-related financial hardship. It is theory-informed, clinic-based, aligned with patient preferences, and has been developed following preliminary qualitative studies and stakeholder input. By design, it will provide prospective evidence on the potential benefits of financial navigation on patient-relevant cancer outcomes. The CAFÉ trial’s strengths include its broad inclusion criteria, its equity-focused sampling plan, its novel intervention developed in partnership with clinical and operations stakeholders, and mixed methods secondary analyses related to intervention dose offered and dose received. The resulting analytic dataset will allow for rich mixed methods analysis and provide critical information related to implementation of the intervention should it prove effective.

**Trial registration:**

ClinicalTrials.gov NCT05018000. August 23, 2021.

## Administrative information

Note: the numbers in curly brackets in this protocol refer to SPIRIT checklist item numbers. The order of the items has been modified to group similar items (see http://www.equator-network.org/reporting-guidelines/spirit-2013-statement-defining-standard-protocol-items-for-clinical-trials/).

**Table:** 

Title {1}	CAFÉ: clinic-based intervention to address financial hardship for people with cancer
Trial registration {2a and 2b}.	ClinicalTrials.gov Identifier: NCT05018000. https://clinicaltrials.gov/ct2/show/record/NCT05018000
Protocol version {3}	Version 1, August 18 2021.
Funding {4}	National Institutes of Health, National Cancer Institute R01CA237322
Author details {5a}	Kaiser Permanente Washington Health Research Institute, Seattle WA (co-authors NBH, MLA, JE, RG, DAK, CL, CN, AR LS, LT) University of California San Diego (co-author MPB) Kaiser Permanente Northwest Center for Health Research, Portland Oregon (co-authors AFP, JD, EK, BL, CM, JR, JLS)
Name and contact information for the trial sponsor {5b}	Erica Breslau, PhD, National Cancer Institute breslaue@mail.nih.gov
Role of sponsor {5c}	The sponsor has no role in the study design, collection, management, analysis, or interpretation of data; writing of the report; or the decision to submit the report for publication.

## Introduction

### Background and rationale {6a}

As costs of cancer care in the USA have risen over time [1, 2], so has the burden of out-of-pocket (OOP) costs [3, 4] and indirect costs such as travel, employment changes, and caregiver costs [5–8]. These cumulative costs pose increased financial risk for people diagnosed with cancer. Despite a patient’s health insurance status, financial hardship from cancer care is prevalent: 47–49% of cancer survivors report financial hardship and 12–62% of cancer survivors report debt due to treatment costs [9]. Financial hardship is associated with decreased treatment initiation and adherence [10, 11], poor symptoms and quality of life [12, 13], and increased mortality risk [14], so preventing or mitigating its effects is a clinical imperative.

Integration of cost of cancer care information into conversations between patients and clinicians can optimize medical decision-making and reduce the risk of financial hardship [2, 15, 16], and is consistent with high-quality cancer care [17, 18] and patient preferences [19–24]. Patient understanding of OOP costs can assist with planning and budgeting and can facilitate early connection with financial support services that may help to mitigate the financial burden of cancer care [25, 26]. Yet, less than one in five patients report having cost discussions [11, 17, 27]. Consequently, many patients are uninformed about the costs of their cancer care [20] and face unexpected OOP costs [28], with important consequences for material (e.g., debt), psychological (e.g., cost-related distress), and behavioral (e.g., treatment adherence) financial hardship [9, 29–31].

There is an urgent need for evidence-based interventions on how to prevent or mitigate financial hardship for people with cancer [32, 33]. While the extent of financial hardship as a toxicity of cancer care is increasingly well-documented, there is limited evidence to date as to what types of interventions can mitigate or prevent financial hardship due to cancer care. Policy-, societal-, and organizational-level interventions, such as those focused on bending the curve of rising health care costs or improving price transparency to ordering providers are all needed, but these may take a long time or show limited effect [9, 29–31]. In the meantime, patients continue to need assistance navigating, managing, and anticipating out-of-pocket costs, and patient- and team level-interventions such as the Cancer Financial Experience (CAFÉ) study intervention may hold promise for this purpose.

An increasing body of observational evidence suggests that communication about financial concerns and out-of-pocket (OOP) costs early in the treatment trajectory and in partnership with the care team could help to prevent or lessen financial hardship. This type of communication could be delivered through patient navigation programs and is consistent with both patient and care team preferences. However, to date, there is no evidence from randomized trials about whether financial navigation during the active treatment period reduces financial hardship. Further, no intervention-based studies have provided evidence on the most effective ways to mitigate cancer-related financial hardship.

### Objectives {7}

Our specific objectives are:

- Objective 1: Compare self-reported financial hardship between patients receiving a financial navigation intervention versus a comparison group at 12-month follow-up.
- Objective 1a: Compare self-reported health-related quality of life between patients receiving a financial navigation intervention versus a comparison group at 12-month follow-up.
- Objective 2: Compare health service use between patients receiving to a financial navigation intervention versus a comparison group at 12-month follow-up.
- Objective 3: Assess patient-level factors influencing variability in dose of a financial navigation intervention.

Our primary hypotheses are that the financial navigation intervention will be associated with improved health-related quality of life and reduced financial distress at 12 months.

Analyses for objectives 1 and 2 will assess the impact of the CAFÉ intervention on the primary outcomes of cancer-related quality of life and financial distress (Aim 1) and on health services use (Aim 2).

Analysis for Aim 3 will also include assessment of the incremental benefit of extended proactive intervention (3 cycles) compared to brief proactive intervention (1 cycle).

### Trial design {8}

**Fig. 1:**
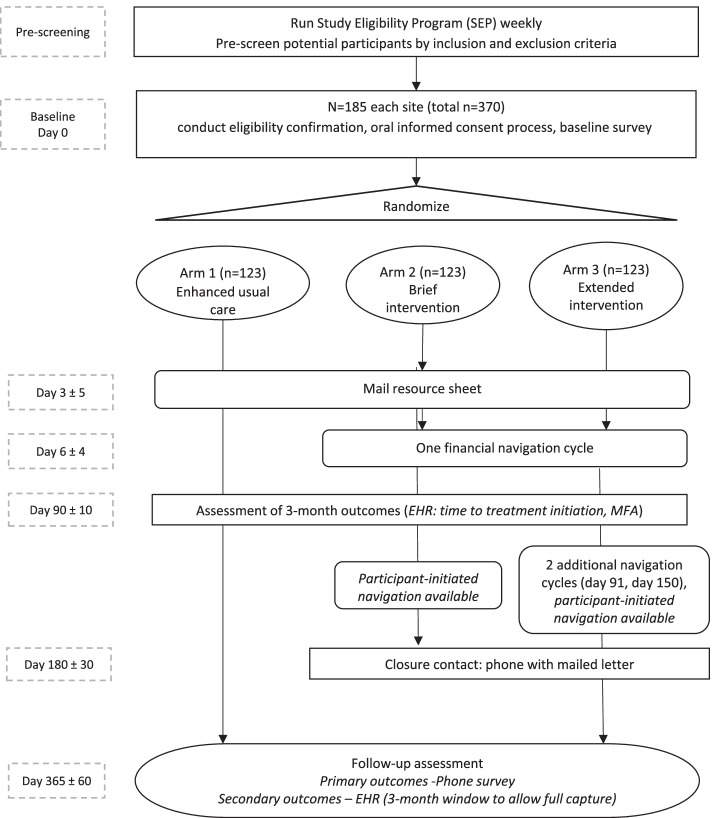
CAFÉ study design overview

The CAFÉ study is a multi-site randomized controlled trial (RCT) with individual-level randomization (Fig. 1). The study has three arms: enhanced usual care (Arm 1); brief intervention (Arm 2); and extended intervention (Arm 3).

The intervention to be studied is a financial navigation intervention. Participants will be offered either brief (one financial navigation cycle, Arm 2) or extended (three financial navigation cycles, Arm 3) financial navigation. The intervention period for both Arms 2 and 3 is 6 months.

The comparison group (Arm 1) will receive enhanced usual care, defined as usual care plus a mailed financial resource sheet describing local and national organizations that serve people with cancer and may address financial concerns.

The original planned study was a clinic-level RCT with stepped wedge design. However, this design could be compromised in the case of COVID-19 related clinic closures, and other COVID-19 related time-trends affecting clinical care. Switching from the clinic-level stepped wedge design to an individual-randomized 3-arm trial protects against these threats to study validity. In addition, we adapted the CAFÉ intervention to be delivered remotely (phone), rather than at in-person visits, further protecting against COVID-19-related changes to clinical care. We chose a 3-group design to assess the impact of the study intervention at two different offered doses (Arms 2 and 3).

## Methods: participants, interventions, and outcomes

### Study setting {9}

The setting for the CAFÉ study is the medical oncology and radiation oncology clinics at two integrated health systems: Kaiser Permanente Washington (KPWA) in Seattle, WA, and Kaiser Permanente Northwest (KPNW) in Portland, OR. We partnered with oncology leadership and business services in both regions to implement the study.

### Eligibility criteria {10}

Inclusion criteria:

- KPNW and KPWA members with a new cancer diagnosis (within the past 120 days from identification date)
- 18+ years of age (based on age at time of identification date by the study eligibility program)
- Visit to CAFÉ clinic/department within two weeks of identification date
- Current, living member as of identification date
- Continuous enrollment at least 6 months prior to identification date
- English speaker for KPWA; English or Spanish speaker for KPNW

Exclusion criteria:

- On do-not contact or no chart review list
- Cancer diagnosis is for non-melanoma skin cancer
- Cancer diagnosis is for a benign or in situ tumor
- Hospice encounter in past year
- Self or household member has already enrolled or completed participation in CAFÉ pilot study or main trial.
- Unable to complete survey

### Who will take informed consent? {26a}

Trained study staff will conduct oral consent and administer the baseline survey by phone, ideally in the same conversation.

### Additional consent provisions for collection and use of participant data and biological specimens {26b}

This trial does not involve collecting biological specimens for storage.

## Interventions

### Explanation for the choice of comparators {6b}

We selected “enhanced usual care” following consultation with both health system and clinical partners as the comparison group. This was to ensure all participants at both study sites receive some information resources about available financial support services. This takes the form of a brief print resources listing available KP and external resources who provide financial resources support to people with cancer. All participants in the study have access to usual care services within the health plan in the form.

### Intervention description {11a}

CAFÉ is a financial navigation intervention designed to provide proactive, personalized, and ongoing support and referrals, including the facilitation of estimates of out-of-pocket costs for the current treatment episode. Several preliminary studies by our study team helped inform the intervention components [25, 26, 34–37]. Further, we conducted a brief pilot of the intervention in 2020, which led to the addition of hospice care as an exclusion criterion, minor adjustments to the study REDCap database, and clarification of recruitment, withdrawal, and intervention procedures.

Broadly, a CAFÉ financial navigator provides support and resources to people with cancer during active cancer treatment. Specifically, the CAFÉ navigator provides the following intervention components: (1) assessment of patient financial concerns according to one or more expected navigation pathway. These pathways are informed by our preliminary work [25] and include acute financial need or inability to pay for household expenses, deciding between care options depending on cost, and uncertainty around out-of-pocket costs and/or when patients will need to pay. (2) Proactive, personalized support, referrals, and navigation to internal and external resources as needed to resolve financial concerns.

The proposed mechanisms of action for financial navigation to influence the outcomes of cancer-related financial hardship and health-related quality of life include:

- Increased affordability of cancer care through connection with financial assistance, payment plan, selection of more affordable treatment plan, and/or increased understanding of upcoming out-of-pocket costs
- Patient has a more patient-centered experience and improved trust with health care system

**Fig. 2:**
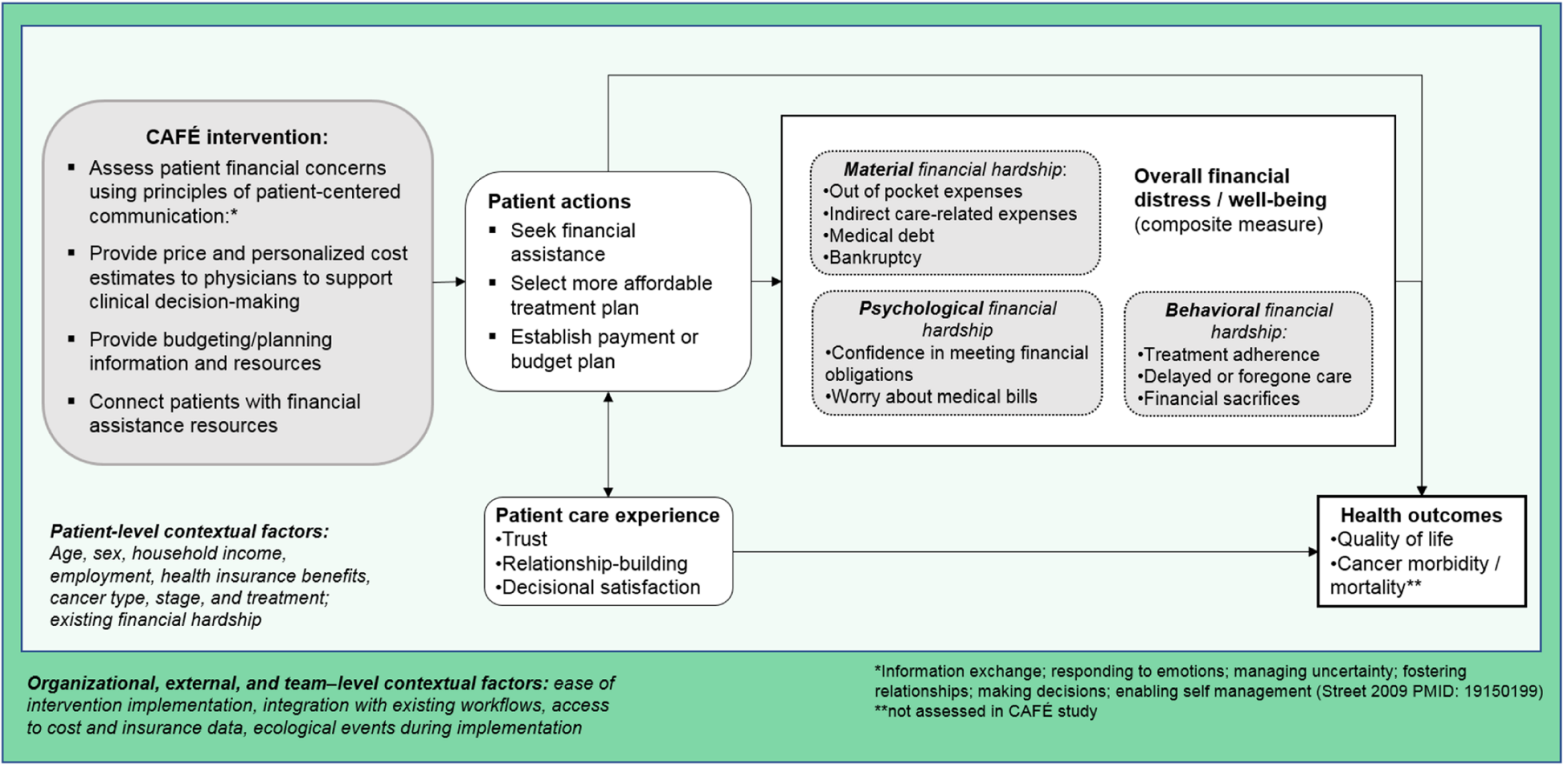
CAFÉ study conceptual model

We developed this novel, intervention building on pilot work and human-centered design methods in partnership with the participating oncology clinic staff and leadership and KPNW and KPWA business services. CAFÉ is founded on the model of patient-centered communication in cancer care (Fig. 2). We chose this conceptual foundation because of the inherent information-exchange function of cost of care communications, rather than a focus on health-related behavior change inherent to other theoretical models. Further, this model provides pathways through which communication can influence health outcomes, either directly through the provision of information and support that can lead to improved health outcomes, or indirectly through trust- and relationship-building and decision satisfaction [38–40].

### Intervention administration

All CAFÉ intervention activities will be administered by trained study interventionists (CAFÉ navigators. Intervention activities will be administered by phone with secure message or mail follow-up.

Intervention conditions include:

- Arm 1 (Enhanced usual care): Mailed financial resource sheet; no contact from CAFÉ navigators
- Arm 2 (Brief intervention, 6 months duration): Arm 1 activities plus one study-initiated financial navigation cycle, participant- or clinician-initiated navigator services available, closure contact
- Arm 3 (Extended intervention, 6 months duration): Arm 2 activities plus 2 additional study-initiated financial navigation cycles (3 total), participant- or clinician-initiated navigator services available, closure contact

One intervention cycle of financial navigation is defined as the following (the duration of each cycle is expected to vary according to participant concern):

1. Financial concerns assessment visit proactively initiated by financial navigator to assess participant’s financial questions and concerns related to cost of care
2. Personalized resources and referrals specific to participant concerns identified during visit
3. Ongoing proactive navigation to provide follow-up around any concerns identified during the visit; expected to vary according to participant needs
4. The end of a cycle will be determined by the CAFÉ navigator, who will record in the study REDCap database.

Participant-initiated or clinician-initiated navigation: During the 6-month intervention period, care teams and participants randomized to Arm 2 and Arm 3 are encouraged to contact the CAFÉ navigator directly as needed during the 6-month intervention period.

Closure contact: Participants in both Arm 2 and Arm 3 will receive a final outreach from the CAFÉ navigator at the end of the 6-month intervention period to inform the participant that their participation in the intervention has concluded. Navigators will attempt to reach the participant by phone; if not reached, the navigator will mail a closure letter.

Coordination with care teams: CAFÉ study investigators worked closely with oncology and health system operations and business leadership at both study sites to design and launch the intervention. Study investigators made presentations to site oncology care teams before the trial launch, and created and shared a one-page information sheet about the trial in the format of “huddle cards” used in typical current practice at both sites.

To maintain communication, CAFÉ navigators inform each intervention participant’s care team via secure staff message of the participant’s participation in the CAFÉ study (Arm 2 or Arm 3). The care team is invited to contact the navigator on behalf of the study participant’s care at any time during the 6-month intervention period.

At the close of each participant’s intervention period, the CAFÉ navigators communicate intervention closure to the participant’s care team via secure EHR message.

### Criteria for discontinuing or modifying allocated interventions {11b}

Participants are free to withdraw from participation in the study (both intervention and outcome assessments) at any time upon request. Participants or relatives/caregivers can inform either the CAFÉ navigators or call the study team as provided in the study information sheet. In this instance, all data flow will stop for that participant (survey assessments, EHR), but data collected will be retained.

The date and reason for participant discontinuation of intervention activities or withdrawal from the study and date of withdrawal will be recorded on the study REDCap database.

Participants who provide consent and are randomized, and subsequently withdraw, or are discontinued from the study, will not be replaced.

A participant will be considered lost to follow-up if we cannot reach them for the final survey assessment. The study team will attempt to contact the participant up to 5 times over 30 days, leaving up to 3 messages, before assigning the participant as lost to follow-up. For participants lost to follow-up, the study team will retain EHR and survey data.

### Strategies to improve adherence to interventions {11c}

CAFÉ navigators make up to 6 call attempts to reach a participant for each assessment visit, then send an “unable to reach” letter with our contact information and encouragement for them to reach out if they are still interested in participating.

### Relevant concomitant care permitted or prohibited during the trial {11d}

There are no restrictions regarding concomitant care during the trial. No alteration of usual care pathways is required for implementation of activities for any of the three trial arms (enhanced usual care; brief financial navigation; extended financial navigation).

### Provisions for post-trial care {30}

All study participants will continue to receive cancer care as determined through clinical shared decision making with their clinical care team.

### Outcomes {12}

Primary and secondary outcomes for the CAFÉ study are shown in Table 1. Primary outcomes include participant-reported financial distress and cancer-related quality of life at 12 months. Secondary outcomes include material, behavioral, and psychological financial hardship; global quality of life; patient-centered communication, and delayed or foregone care as measured by health service use at 12 months.

**Table 1:** CAFÉ study endpoints

Objectives	Endpoints assessed at 12 months	Justification for endpoints
**Primary**		
1. To compare self-reported financial hardship between patients receiving the CAFÉ intervention versus a comparison group at 12-month follow-up.	Financial distress [InCharge Financial Distress/Financial Well-Being Scale] [41]	Financial hardship is an established social determinant of health.
1a: To compare self-reported health-related quality of life between patients receiving the CAFÉ intervention versus a comparison group at 12-month follow-up.	Health-related quality of life [cancer-specific: FACT-G7] [42]	Cancer-related financial hardship is directly associated with multiple adverse health outcomes (e.g., quality of life; pain, symptom burden, mortality).
**Secondary**		
2. Compare health service use between patients receiving the CAFÉ intervention versus a comparison group at 12-month follow-up.	Survey: Material, behavioral, and psychological financial hardship Medical Expenditure Panel Survey (MEPS) items [43] Global quality of life PROMIS Global health [44–47] Patient-centered communication: Patient Assessment of cancer Communication Experiences (PACE) [48] (EHR) Delayed or foregone care assessed by treatment initiation^a^ and time to treatment initiation^a^ Use of KP medical financial assistance services^a^; total out-of-pocket expenditure; account delinquency	The CAFÉ intervention could facilitate early identification of patient affordability concerns and care plan adjustments; improve access to financial planning resources or payment plans; and by connection to referrals and resources for acute financial distress.
3. Assess patient-level factors influencing variability in dose of the CAFÉ intervention.	Intervention dose received (Arm 2 vs Arm 3)	Estimation of ideal dose will be important for future implementation of the intervention.
**Exploratory/covariates**		
Exploratory	Difficulty paying bills, ongoing financial stress, medication reduction due to cost, food insecurity [49, 50]; difficulty living on current income [51]; decisional regret; decision satisfaction	The CAFÉ intervention may directly impact these endpoints but analyses are exploratory.
Covariates	Socioeconomic variables; clinical variables; existing financial burden; insurance characteristics (health insurance type; maximum out-of-pocket limit; deductible limit; co-Insurance or co-payment)	

^a^Health service use outcomes measured via EHR will be assessed at both 3 months and 12 months

### Participant timeline {13}

An individual’s participation duration is 12 months. A participant is considered to have completed the study if they have completed the baseline assessment, at least 1 intervention cycle (Arms 2 and 3 only; defined as one financial navigation visit plus associated follow-up contacts to resolve concerns identified in the visit), and the 12-month follow-up assessments (Table 2).

**Table 2:** CAFÉ study participant timeline

	Pre-screening	Day 0	Day 7	Day 10	Day 90	Day 91	Day 150	Day 180	Day 365
EHR review eligibility	X								
Informed consent		X							
Randomization		X							
Baseline data collection		X							
**Outcome evaluation**									
Participant-reported outcomes									X
EHR outcomes					X				X
**Intervention activities**									
Enhanced usual care activities		X							
Navigation visit (Arm 2)				X					
Navigation visit (Arm 3)				X		X	X		
Participant-initiated navigation (Arm 2, Arm 3)				X	X	X	X		
Closure contact (Arm 2, Arm 3)								X	
Adverse events reporting		X	X	X	X	X	X	X	X

### Sample size {14}

The study will enroll 185 participants at each site (total *n*=370; approximately 123 per randomization group). Primary outcome measures for Objective 1, are assessed via survey at 12 months. Assuming 20% attrition, the analytic sample size for Aim 1 outcomes will be 296 patients (*n*=99 per group). With a type 1 error rate of 0.05 (two-sided), we will have 80% power to detect effect sizes of Cohen’s *d* = 0.40 for continuous outcomes. For the primary outcome of financial distress, this translates to a least detectable difference of 0.96 on the InCharge financial distress scale (10-point measure) for pairwise comparisons between randomization groups, assuming a standard deviation of 2.4 [41]. For the binary financial hardship measures (secondary outcomes), the least detectable difference depends on the proportion reporting financial hardship in the comparison group. We have 80% power to detect at 17.4% difference in financial hardship if the reported rate is 35% in the comparison group, corresponding to a rate of 17.6% among intervention patients (pairwise comparison of randomization groups). If the rate among comparison patients is much lower at 15%, the least detectable difference is 11.4%.

Based on our estimated sample size and a standard deviation of 5.0 as reported in the measure development work establishing the validity and reliability of the FACT-G7 in ambulatory cancer patient population [42], we estimate that we will be able to detect a 2.0-point difference in health-related quality of life (HR-QOL) between intervention and control groups. Since a 2 to 3 point difference on the FACT-G7 is considered clinically meaningful [42], we will be able to detect clinically meaningful changes on the FACT-G7 during the CAFÉ study.

### Recruitment {15}

We will identify eligible participants using the CAFÉ study eligibility program (SEP). We have developed the SEP specifically to identify patients eligible for the CAFÉ study using electronic health record (EHR) data. Patients are eligible if they have a new cancer diagnosis and an initial visit to a participating CAFÉ study clinic following their diagnosis [43] in addition to meeting the other inclusion criteria. We have validated the SEP against manual chart review as the gold standard.

#### Equity-informed sampling

We established a goal that individuals from historically under-represented and under-studied populations will comprise up to 50% of the enrolled participants. We did this to maximize opportunities to identify unique insights from a more diverse study population, as well as to be responsive to the ethical principle of justice by promoting greater fairness in access to equal opportunity for participation and the distribution of the benefits and risks of the research.

Using EHR data, we will use the following four enrichment characteristics to identify individuals from populations historically under-represented and under-studied in clinical trials: non-White race; Hispanic ethnicity; Spanish as a preferred spoken language (KPNW only); and Medicaid beneficiary status. We will prioritize for recruitment of individuals who meet any of the four enrichment characteristics.

#### Manual chart review

Our initial SEP design and validation identified some areas where manual review is necessary to confirm an individual’s eligibility. Thus we include selective manual verification step to confirm participant eligibility for individuals whose CAFÉ-qualifying cancer diagnosis meets the following criteria: cancer type is the “Other” CAFÉ study cancer group (i.e., not breast, colorectal, lung, or prostate cancer); diagnosis is suggestive of a metastatic cancer diagnosis; or noted prior cancer diagnosis within 10 years different than the CAFÉ-qualifying cancer diagnosis (i.e., a new primary tumor that is diagnosed within 10 years of a prior primary tumor at a different site).

#### Recruitment procedures

To recruit participants, we will mail advance letters and a study information sheet. The information sheet will describe the study, include instructions on how to opt out, and state that we will call to invite them to participate in the study, obtain consent, and conduct the baseline survey.

## Assignment of interventions: allocation

### Sequence generation {16a}

After completion of baseline questionnaire, participants will be randomized via a computer-generated randomization scheme developed by a study biostatistician in a 1:1:1 ratio to study groups (Enhanced usual care, Brief intervention, or Extended intervention) stratified by site (KPWA; KPNW), and cancer type (breast, lung, prostate, colorectal, other). Our recruitment goal is to enroll a sample that reflects the distribution of cancer types in the underlying clinical population, and to enroll an equal number of participants at each study site. As such, we will flex the sampling during the recruitment period to meet this goal.

We will employ random blocks of sizes 3 and 6 to ensure balance of groups over time as well as blinding of study team to next randomization assignment. The biostatistician will keep the randomization file in a secure folder only accessible to the biostatisticians and programmer.

### Concealment mechanism {16b}

The study biostatistician will provide the study programmer with the randomization scheme within specified strata. The programmer will develop a program that will only allow participants to be randomized once they consent and complete the baseline questionnaire. This method ensures that treatment allocation cannot be changed after randomization.

### Implementation {16c}

The study biostatistician will generate the allocation sequence. Study staff will enroll participants. After consenting and collecting baseline data from the participant, the study interviewer will press a button within the REDCap database and the appropriate group assignment will be revealed.

## Assignment of interventions: blinding

### Who will be blinded {17a}

This is an unmasked trial for participants and study interventionists, as it is not possible to provide the intervention without revealing the study group.

The survey administrators will be blinded to study group assignment 12-month survey, where primary and secondary self-reported outcomes will be collected. Baseline data collection will be conducted before randomization.

No members of the core study team, including PI’s, biostatisticians, programmers, or interventionists will have access to unblinded data. Study biostatisticians and programmers may access data (blinded by study arm) for quality control and study progress reporting purposes, as necessary.

### Procedure for unblinding if needed {17b}

Partial unblinding (study group assignment to “any intervention” vs. “enhanced usual care”) will occur to biostatisticians and programmers only at the time of analysis of 3-month EHR outcomes. Otherwise, unblinding will only occur once the final 12-month outcomes dataset is assembled and locked.

## Data collection and management

### Plans for assessment and collection of outcomes {18a}

All recruitment and participant-reported outcomes data is collected and stored in a bespoke REDCap study database developed by the study team.

#### Survey

Participant-reported outcomes and covariate data will be collected via telephone survey at baseline and 12 months. Trained survey administrators will conduct all surveys using IRB-approved survey instruments. Survey administrators will enter survey data directly into the study REDCap database.

#### Administrative data

EHR-based data will be collected at baseline, 3 months, and 12 months, including covariate and outcome data. Data to be collected will be consistent across study sites. Data sources include virtual data warehouse (VDW) data elements developed by the Healthcare Systems Research Network (HCSRN) and EPIC® Clarity.

#### CAFÉ navigator notes and closure questions

CAFÉ navigators will use the study REDCap database to conduct all intervention activities. Navigators are trained in the use and administration of the database for both intervention administration and entering of research data that will allow assessment of participant dose received and intervention fidelity and adherence. Fields in the database are both open text (e.g., to describe resources provided to a participant) and discrete fields (e.g., completion of an assessment visit; type of financial concern addressed in visit).

Navigators will, at each intervention participant’s closure call, ask four brief questions about the participant’s experience with the intervention and make field notes summarizing the participant’s answers to these questions in an open text field.

Copies of surveys and data dictionaries are available on request to the principal investigators.

### Plans to promote participant retention and complete follow-up {18b}

Study staff collecting survey outcomes will attempt to reach participants up to 5 times for baseline and follow-up data collection. Participants will receive an incentive of $10 for completion of the baseline survey $25 for completion of the follow-up survey.

### Data management {19}

The study participants’ contact information will be securely stored at each clinical site for internal use during the study. At the end of the study, all records will continue to be kept in a secure location for as long a period as dictated by the reviewing IRB, Institutional policies, or sponsor/funding agency requirements.

Study participant research data, which is for purposes of statistical analysis and scientific reporting, will be transmitted to and stored at KPWA and KPNW. This will not include the participant’s contact or identifying information. Rather, individual participants and their research data will be identified by a unique study identification number. The study data entry and study management systems will be secured and password protected. At the end of the study, all study databases will be de-identified and archived.

### Confidentiality {27}

Data that could be used to identify a specific study participant will be held in strict confidence within the research team. No personally identifiable information from the study will be released to any unauthorized third party without prior written approval of the sponsor/funding agency. All research activities will be conducted in as private a setting as possible.

### Plans for collection, laboratory evaluation, and storage of biological specimens for genetic or molecular analysis in this trial/future use {33}

This trial does not involve collecting biological specimens for storage.

## Statistical methods

### Statistical methods for primary and secondary outcomes {20a}

#### Analysis of the primary endpoint(s)

The trial has two primary outcomes, financial distress, and cancer-specific health-related quality of life (HR-QOL). Both of these outcomes are continuous measures, which will be assessed via survey at 12 months after randomization. Financial distress will be measured using the InCharge Financial Distress/Financial Well-Being scale, and HR-QOL will be measured by the cancer-specific FACT-G7 scale.

Each outcome will be evaluated in a separate model. We will use linear regression models to estimate group means, using generalized estimating equations (GEE) with an independent working correlation structure and robust covariance estimation. The dependent variable will be the 12-month study outcome (financial distress, or HR-QOL), and the independent variables will be indicator variables for randomization group. Models will account for the clustering of patients within oncology departments. Estimates of intervention effects will be presented as differences in group means, with 95% confidence intervals. We will fit unadjusted models, and models adjusting for covariates, and models adjusting for measures imbalanced by randomization group, related to missing outcome data, and baseline measures of the outcome (where appropriate).

#### Analysis of the secondary endpoint(s)

Analysis of secondary outcomes that are continuous such as out-of-pocket expenditure will follow the same analytic approach outlined for the primary outcomes. For binary outcomes, such as initiation of treatment, use of financial services (yes/no), or account delinquency, we will use modified Poisson regression models. Models will use a log link function estimated with GEE, with an independent working correlation structure and robust covariance estimation, and will account for clustering by oncology department. Intervention effects will be presented as the relative risk of the outcome in each of the intervention groups relative to Usual Care, with 95% confidence intervals.

To assess the short-term impact of financial navigation on health service use and treatment outcomes, we will conduct analysis of several secondary outcomes at 3 months. These include (1) medical financial assistance (MFA) application (yes/no) [Secondary: MFA application approved or denied; type of MFA award received] and (2) initiation of treatment (yes/no) [Secondary: time-to-initiation of treatment; type of treatment]. Assessment of 3-month outcomes will follow the general approach outlined for 12-month primary and secondary outcomes, except that the intervention group will be a binary variable. Group 2 (Brief intervention) and Group 3 (Extended intervention) participants will be combined for analysis, and effect estimates will compare this combined intervention group to usual care.

### Interim analyses {21b}

No interim analyses are planned.

### Methods for additional analyses (e.g., subgroup analyses) {20b}

#### Mixed methods analysis: patient-level factors associated with intervention dose received

The optimal dose of financial navigation needed to impact financial hardship and health-related quality of life is not well understood and has substantial implications for future implementation of financial navigation interventions. By design, intervention participants (Arms 2 and 3) can initiate contact with CAFÉ navigators any time during the 6-month intervention period, and so can receive additional navigation services beyond the minimum specified in the protocol. We therefore expect that the total dose received of the CAFÉ intervention to vary between participants based on individual needs; thus, our third study aim involves mixed methods analyses of patient-level factors associated with the intervention dose received.

Our conceptual definition of dose includes several components: the total quantity of financial navigation cycles offered and received; participant engagement/participation in the intervention; and appropriateness of the CAFÉ intervention for addressing participant financial questions and concerns. Further, we distinguish conceptually between the intervention dose offered per the protocol and dose received: the total number of navigation cycles that each intervention participant receives (Table 3).

**Table 3:** CAFÉ study dose-related constructs

Construct	Definition
Dose offered (aka adherence per protocol)	- One financial navigation cycle over 6 months (Arm 2 brief intervention participants) - Three financial navigation cycles over 6 months (Arm 3 extended intervention participants)
Navigation cycle	One financial assessment visit between navigator and participant plus associated follow-up until concern is addressed
Ad hoc cycle	Financial navigation cycle initiated outside of protocol cycles (spontaneous contact initiated by participant, family member, or clinical team) to disclose a new concern
Total dose received	Total number of navigation cycles completed by the participant (protocol cycles plus extra cycles)
Cycle initiator	Whether intervention cycle was per protocol; participant-initiated; or clinician-initiated
Navigation pathway	Concerns addressed in each intervention cycle (acute financial needs; planning/budgeting for future expenses; need for cost information to inform clinical decision-making; or low touch)

We plan three quantitative analyses related to dose (a) intervention effects of the randomized dose offered, (b) predictors of high versus low dose received, and (c) differences in study outcomes controlling for dose received. We will conduct descriptive and multivariable analyses for each of these analyses, including sociodemographic and other covariates as well as characteristics of the intervention cycles (e.g., cycle initiator and financial concerns pathways).

Qualitative analyses will include thematic content analysis of navigator notes from the study REDCap database, including the four brief questions about the participant’s experience with the intervention asked at the final closure contact. Analysts will conduct qualitative content analysis, with the goal of providing insight into participant engagement with the intervention, as well as reports of the appropriateness or fit of the intervention with participant needs.

Our mixed methods approach will use a sequential explanatory design with triangulation protocol [44, 45]. We will collect quantitative and qualitative data relatively independently but iteratively where appropriate, with a series of triangulation analysis meetings to discuss convergence, complementarity, and discrepancy of findings. For example, if quantitative analysis identifies a specific group of participants with a very high intervention dose received; qualitative analysts might conduct a focused analysis to explore the fit of the intervention for that group.

### Methods in analysis to handle protocol non-adherence and any statistical methods to handle missing data {20c}

The investigators and data managers will monitor data collection process on a weekly basis and ensure a minimal amount of loss to follow-up in the study. All analyses will be conducted following a modified intent-to-treat approach, including all individuals randomized regardless of their engagement with, or exposure to the intervention. Individuals with missing outcome data will not be included in the primary analysis. We will conduct an analysis assessing factors related to missing outcome assessment. We will fit logistic regression models with missing primary outcome (yes/no) as the binary outcome in the logistic regression model, and baseline characteristic variables as independent variables, to determine predictors for missingness. Baseline covariates that are significantly (at 0.10 significance level) associated with missingness from this model will be adjusted in all models to ensure the validity of a missing-at-random (MAR) assumption. If loss to follow-up is above 15%, we will employ appropriate statistical techniques to address missing data issues. We will consider multiple-imputation analyses, or inverse probability weighting to account for missingness.

### Plans to give access to the full protocol, participant-level data, and statistical code {31c}

Full protocol, datasets, and statistical code may be requested by contacting the study principal investigators. After the study is completed, the de-identified, archived data will be transmitted to and stored at KPWA for use by other researchers including those outside of the study.

## Oversight and monitoring

### Composition of the coordinating center and trial steering committee {5d}

The study team will manage the day-to-day monitoring of the trial. The team consists of the principal investigators, co-investigators, biostatisticians, programmer analysts, project managers, interventionists, and qualitative researchers. In addition, a patient advisory group for the study provides consultation and review of study documents.

### Composition of the data monitoring committee, its role, and reporting structure {21a}

A data monitoring committee will not be needed, as the financial navigators are not blinded and can respond and/or report any adverse events for this low-risk intervention.

### Adverse event reporting and harms {22}

We anticipate this research involves no more than minimal risk to participants. Potential adverse events include breach of confidentiality; discomfort at some survey questions or intervention interactions; or perceived or actual stigma related to disclosure of financial hardship. The study team will report any observed adverse events to the principal investigators. The principal investigators will discuss all such events with the team and the IRB and take action(s) as appropriate. Summary reports of any such adverse events and subsequent actions taken will also be provided to the NIH.

### Frequency and plans for auditing trial conduct {23}

The study team will meet weekly to review the trial activity. At this time no formal audits are planned.

### Plans for communicating important protocol amendments to relevant parties (e.g., trial participants, ethical committees) {25}

The study principal investigators will seek approval from the Kaiser Permanente Washington Human Research Protections Program for any planned modifications.

### Dissemination plans {31a}

This study will comply with the National Institutes of Health (NIH) Public Access Policy, which ensures that the public has access to the published results of NIH funded research. It requires scientists to submit final peer-reviewed journal manuscripts that arise from NIH funds to the digital archive PubMed Central upon acceptance for publication. In addition, every attempt will be made to publish results in peer-reviewed journals. The study team will also disseminate results to internal stakeholders (oncology and business) at both study sites.

## Discussion

The CAFÉ study will provide much-needed early trial evidence on the impact of financial navigation in reducing cancer-related financial hardship. It is theory-informed, clinic-based, aligned with patient preferences, and has been developed following preliminary qualitative studies and stakeholder input. By design, it will provide prospective evidence on the potential benefits of financial navigation on patient-relevant cancer outcomes. The CAFÉ trial’s strengths include its broad inclusion criteria, its equity-focused sampling plan, its novel intervention, and mixed methods secondary analyses related to intervention dose offered and dose received. The resulting analytic dataset will allow for rich mixed methods analysis and provide critical information related to implementation of the intervention should it prove effective.

Due to the COVID-19 pandemic, we adjusted the original design, which included a switch to an individual-level randomized trial design and a switch from in-person to telephone-based navigation intervention. We tested these new procedures in a small pilot before proceeding with the current protocol. Changes we made based on this pilot included procedures for including care partners in financial navigation encounters and minor adjustments to our recruitment and intervention procedures. Importantly, following the pilot we also conducted a health equity-informed review of all trial procedures, guided by health equity frameworks in the literature and our institution’s internal equity framework. This review was extremely helpful; changes included the addition of procedures for participants with hearing or visual impairments and the altering of our study eligibility sampling program to maximize representation of participants from historically under-represented and under-studied populations.

Potential limitations include possible co-occurring ecologic changes external to the intervention, such as policy or organizational changes, that could impact the intervention’s impact. Based on our strong partnerships with oncology clinic staff, we expect to implement the intervention as intended, but we will document any such changes during the trial period and assess if changes to our analyses are needed. Additionally, although CAFÉ navigators support participants in navigating insurance changes and seeking both internal and community financial support resources, we are not able to alter insurance generosity, patient out-of-pocket charges, or larger health policy with this intervention. The generalizability of the CAFÉ intervention is top of mind, given the integrated health system setting of the trial. We have intentionally designed the intervention to be relevant for all cancer types and stages, which will provide valuable data related to the value of cancer financial navigation programs in real-world settings.

## Trial status

The trial is enrolling participants as of August 10, 2021. Enrollment is expected to be completed in February 2023.

## Data Availability

This study will comply with the NIH Data Sharing Policy and Policy on the Dissemination of NIH-Funded Clinical Trial Information and the Clinical Trials Registration and Results Information Submission rule. As such, this trial will be registered at ClinicalTrials.gov, and results information from this trial will be submitted to ClinicalTrials.gov. In addition, every attempt will be made to publish results in peer-reviewed journals. Data from this study may be requested from other researchers by contacting study principal investigator.

## Abbreviations

CAFÉ: Cancer Financial Experience
EHR: Electronic health record
GEE: Generalized estimating equations
HR-QOL: Health-related quality of life
KPNW: Kaiser Permanente Northwest
KPWA: Kaiser Permanente Washington
MAR: Missing at random
MFA: Medical financial assistance
NIH: National Institutes of Health
OOP: Out of pocket
SEP: Study eligibility program
